# Introducing a Method of In Vitro Testing of Different Anchoring Systems Used for Female Incontinence and Prolapse Surgery

**DOI:** 10.1155/2013/401417

**Published:** 2013-12-22

**Authors:** Ralf Anding, Ruth Tabaza, Manfred Staat, Eva Trenz, Philipp Lohmann, Uwe Klinge, Ruth Kirschner-Hermanns

**Affiliations:** ^1^Department of Neuro-Urology, University Hospital, Rheinische Friedrich-Wilhelms-University, 53127 Bonn, Germany; ^2^Institute of Bioengineering, FH Aachen University of Applied Sciences, Juelich Campus, 52428 Juelich, Germany; ^3^Department of Surgery, University Hospital RWTH Aachen, 52074 Aachen, Germany

## Abstract

*Aims*. To develop a method for in vitro testing to compare different intracorporeal anchoring systems (AS) used, for example, in single-incision slings or vaginal meshes. Intracorporeal fixation needs reliable anchorage systems, which keep the implant in the operative and early postoperative phase in place. Up to now, the impact of the design of current anchor systems and their capability to provide sufficient retention forces is not known. *Methods*. Four AS (“PelFix”, “Surelift”, “TFS”, and “MiniArc”) were evaluated in an animal model and a ballistic gelatine model with pull-out tests. We performed ANOVA with post hoc Bonferroni. Results were presented as mean values whereby a significance level of <0.05 was considered significant. *Results*. The four systems showed significantly different pull-out forces. Depending on mesh structure, size, and form of the AS, mechanical strain resulted in deformation with local peak stresses. Under the condition of form stability, relative differences of pull-out forces did not change in different tissues. *Conclusions*. Reliable testing of different AS in their ability to keep mesh implants in place can be done in animal models and in especially designed ballistic gelatine. These methods of testing will help to modify AS in novel pelvic floor implants.

## 1. Introduction 

Since the introduction of TVT Secur (Gynecare/Johnson&Johnson) in 2006, various devices with self-anchoring systems for use in female pelvic floor surgery have made their way onto the market. This market experienced some disturbances in 2012 when Johnson&Johnson felt obliged to withdraw many of their products due to regulatory affairs that involved TVT Secur. In the face of some uncertainty concerning the future use of mesh devices, we feel that it is important to take a step back and have a closer look at single aspects of custom-made devices. This reversal in thinking can help to detect weak points of mesh kits before using them in clinical trials.

Early dislocation of mesh material is one major risk factor for failure especially when miniaturized meshes are used. First comparative data suggest that the single-incision sling procedure, when correctly placing the sling, leads to nearly equal success rates as conventional suburethral tapes, but with fewer complications [1]. These minimally invasive procedures depend on a reliable intracorporeal fixation with an anchoring system (AS) that prevents dislocation. Since the success of single-incision sling procedures relies on some degree of tension [1, 2], mechanically reliable AS are essential.

The integral theory developed by Petros and Ulmsten in 1993 [3] emphasizes the role of the connective tissue of the pelvic floor muscles and the supporting ligaments in both function and dysfunction, as well as in surgical repair. The concept is based on the analogy of the pelvic floor with a suspension bridge for structure in combination with a trampoline for function [4]. This led to the defect-oriented concept for repairing pelvic floor pathologies. The concept of the “tension fixation system” (TFS) comprises the use of slings to substitute the impaired ligaments, which are inserted under direct vision using polypropylene anchors [5].

The whole concept of the TFS, as well as the SIS, can only work with a reliable AS that ensures stability, particularly during the surgical procedure and in the first weeks after surgery. Several AS have been developed: absorbable patches (TVT Secure), AS with a self-adherent surface (DynaMesh SIS minor) or tapes with minimized anchors such as the “MiniArc” [1]. So far we have been lacking reliable methods to test different types of AS in this regard.

Earlier studies in the field of abdominal hernioplasty examining intra-abdominal pressures and tensile strength of tissues show that the minimum holding force of a textile device should exceed 32 N/cm [6]. According to Cosson et al., vaginal tissue strength is usually lower and very different from one individual to another [7]. In a recent calculation by Ozog et al., the membrane tensions of the female pelvic floor do not exceed 1.7 N/cm [8]. According to Naumann et al., in a review on single-incision slings (SIS), one of the unsolved problems of all minisling systems is to find a way of getting a certain degree of controlled tension on the tape [1, 9]. Thus, the goal of our study is to develop a method to identify the type and material of AS that is most suitable for the use in pelvic floor surgery. We compared AS that are already on the market such as “MiniArc” (by AMS); “Surelift” (by Neomedic); “TFS AS” (by TFS Surgical); and the new system under development, “PelFix” (by FEG).

## 2. Methods

**Figure 1:**
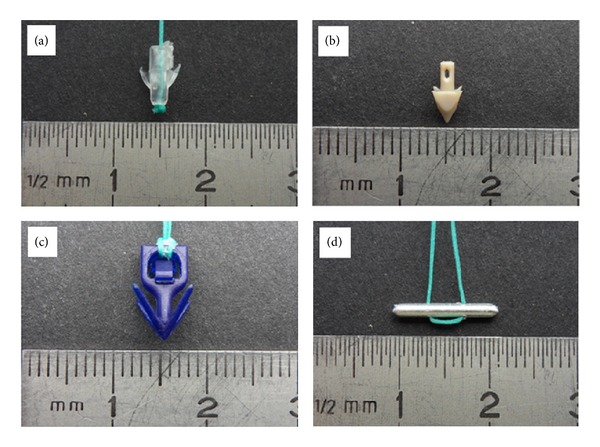
Types and sizes of four different anchoring systems. (a) The “MiniArc” anchor (by AMS) is made of a cylindrical body with two shark-fin-like hooks. (b) The “Surelift” anchor (by Neomedic) is cone-shaped with a cylindrical shaft ending in a squared dome with towering ends at the edges that act as hooks. (c) The “TFS” anchor is equipped with 4 hooks arranged as the four sides of a pyramid. (d) The “PelFix” anchor consists of an ellipsoid that is fixed to a filament. After introduction inside a tube and retracting the filament, the pin turns horizontal and gets hooked in the tissue.

We tested the following four AS: “MiniArc-AMS,” “Surelift-Neomedic,” “TFS-TFS Surgical,” and “PelFix-FEG” (Figures 1(a), 1(b), 1(c), and 1(d)). These products differ not only in their material, with PelFix being made of polyvinylidene fluoride and all others, being made of polypropylene, but also in form and size. All systems comply with the standards set by the ICS/IUGA 2010 [9]. For better comparison, the anchors were separated from the custom made meshes and fastened to identical threads.

### 2.1. Tissue Testing (Ex-Vivo Animal Model)

Actual forces on pelvic floor structures, especially when coughing or straining, can only be estimated. We assume that they are comparable to pressures measured for hernia repair with pressures during coughing at 60 ± 14 mmHg with a maximum of 91 mmHg.

**Figure 2:**
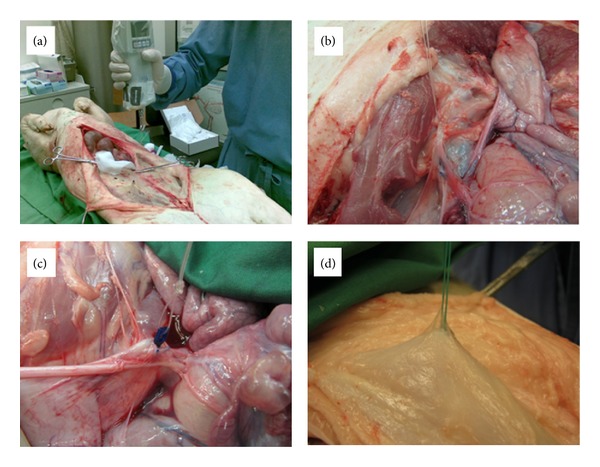
Anchor pull-out tests in different tissues of a pig. (a) Measurement of the holding force in the porcine rectus fascia with the specialized instrument (Sauter FK50, 50 N/0.02 N). (b) Measurement of the holding force in the mesentery. (c) Measurement in the broad ligament. (d) Anchoring system in the porcine rectus fascia.

First, all AS were tested in an animal model. We used young domestic pigs (German landrace) that underwent euthanasia. Their weight ranged from 30 to 40 kg. To compare the different AS, we first measured the extraction force in the porcine pelvic floor three times for each sample. All anchors were tightly attached to a specialized force-measuring instrument (Sauter FK50, 50 N/0.02 N, Figure 2(a)). The forces needed for extraction were measured in Newton (N), and the measurements were limited to a maximum of 50 N as this value is far above the range assumed to be physiologically relevant.

Preparation of the porcine pelvic floor revealed that the strength of the corresponding structures was considerably lower than in humans. Furthermore, certain structures that are present in the female human pelvis such as the arcus tendineus and the sacrouterine ligaments are missing in the animal model. Nevertheless, we started by placing the anchors in the mesentery and the broad ligament (Figures 2(b) and 2(c)), a structure analogous to the cardinal ligament of the human pelvic floor. In addition, we compared the pull-out forces of the four different anchor systems in the rectus fascia with the underlying muscles, which seemed to be more comparable to the strength of the arcus tendineus and the sacrouterine ligaments. After removing the skin of the pig's abdomen, an additional comparison of pull-out forces using rectus fascia was done. For each AS, the testing of the rectus fascia was repeated ten times using slightly different locations (Figure 2(d)).

For statistical analysis, we performed ANOVA with post hoc Bonferroni. The results were presented as mean values whereby a significance level of *P* < 0.05 was considered significant (Table 2).

### 2.2. In Vitro Testing

**Figure 3:**
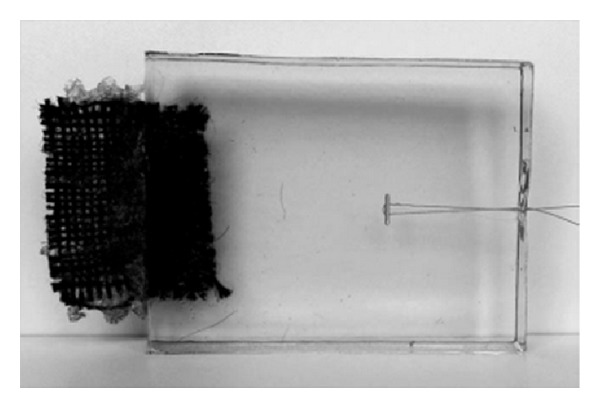
Specimen made of ballistic gelatin with casted-in PF anchor. The fabric on the left side of the specimen is used for proper clamping in the tensile testing machine.

For visualisation of local strain, we adopted a method developed by Staat et al. in 2012 [10] and put the different AS into a cuboid of ballistic gelatine (255-265 Bloom Type A gelatine, Gelita AG, product name: Type Ballistic 3, Figure 3). Clamped into a standardised Zwick/Roell tensile testing machine (Z010/TN2A, load cell type KAP-Z), a tensile test was performed with a preload of 3 N at a cross-head velocity of 10 mm/min and a test velocity of 40 mm/min until failure. The stress profile around the anchor was assessed with a polariscope, consisting of a light source and at least two polarizing filters. In this setting isochromatic lines are points of equal shear stress magnitudes and they are proportional to the tension in the material. Generally speaking, the higher the density of lines in a certain area, the higher the stress gradient. Since the photoelastic constant of the ballistic gelatine has not yet been defined, we limit ourselves to a qualitative analysis.

## 3. Results

For all AS, the extraction forces in the *pelvic floor* were considerably lower than those in the *rectus fascia* (Table 1). Here, none of the AS reached a holding capacity of more than 16 N. In the pelvic floor as well as in the rectus fascia, the “MiniArc” revealed the lowest pull-out force, whereas “PelFix” showed the best fixation in both structures. Forces observed for “MiniArc” and “Surelift” were fourfold higher in the rectus fascia than in the pelvic floor. “TFS” presented the smallest difference in both structures.

In the *rectus fascia*, “PelFix” showed the highest resistance to extraction, significantly more than the other three anchors (*P* < 0.05). “Surelift,” which was kept tight to the tissue, was second (*P* < 0.05) followed by “MiniArc” and “TFS” (Table 2). “MiniArc” and “TFS” both demonstrated low resistance against extraction with mean forces far below 32 N. Although “Surelift” reached fairly high pull-out forces with a mean value of >32 N, this limit was not reached in all measurements. Only the “PelFix” anchor system revealed a constant extraction force above 50 N in all measurements.

**Figure 4:**
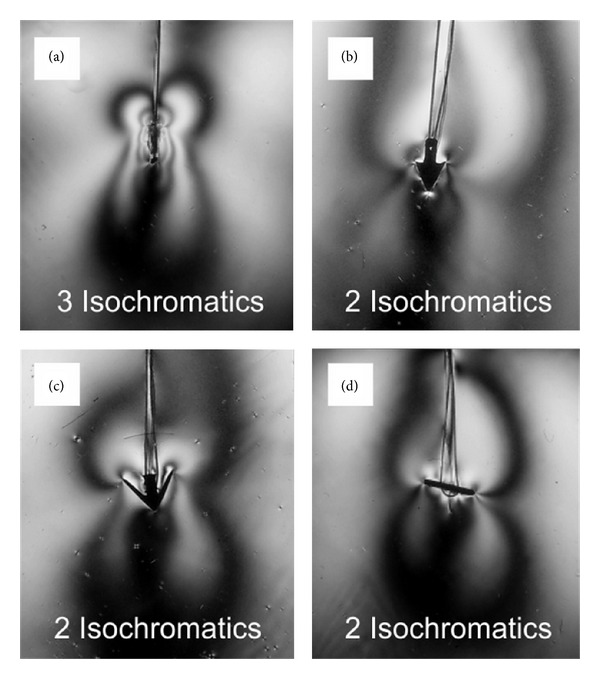
Photoelastic experiments to visualize “stress profiles” of different anchoring systems. (a) Isochromatics of MiniArc anchor. (b) Isochromatics of Surelift anchor. (c) Isochromatics of TFS anchor. (d) Isochromatics of PelFix anchor.

Photoelastic studies with a polariscope helped to visualize the “stress profiles” of the AS and also revealed particular differences in the distribution of local stress among the four anchors (Figures 4(a), 4(b), 4(c), and 4(d)). The “MiniArc” anchor (AMS) showed a higher number of isochromatic fringes under a load of 3 N than the other anchors (an indication for high local stress resulting in low pull-out forces). In the pull-out testing of the different AS, the isochromatic fringes in ballistic gelatine reflect the distribution of the shear stress under a constant load of 3 N. This is already more than 70% of the pull-out force of the weakest anchor.

One can also easily identify the sharp edges of the anchors as the origin of the emerging isochromatic fringes and thus the source of the stress concentration. The “MiniArc” anchor showed at least 50% higher stress under the same load that can be attributed to a 50% lower holding force. The stress magnitudes produced by the other anchors did not differ much so that similar holding forces can be expected for these anchors. The mean values of the holding forces measured in ballistic gelatine are 4.2 ± 0.4 N (*n* = 5) for the “MiniArc” anchor, 7.1 ± 0.8 N (*n* = 5) for the “Surelift” anchor, 8.2 ± 0.8 N (*n* = 4) for the “TFS” anchor, and 7.8 ± 0.9 N (*n* = 5) for the “PelFix” anchor. The standard deviation in the gelatine testing is approximately 5% of the measured values and even higher in the tissue testing.

Corresponding pull-out forces in gelatine and other tissues (*F*/*F*_PelFix_, Table 1) remained constant in relation to PelFix except for “TFS” when tested in the rectus fascia. This can be easily explained by the fact that we observed a strong deflection of the arms of the “TFS” under higher loads. With forces greater than 15 N, the “TFS” anchor lost its structural stability.

In order to additionally compare the pull-out forces of the “TFS” system in the animal model with the data of the gelatine model, we tested the anchor without its adjustment system. We did so because only “TFS” works with this adjustment system, but the adjustment system slipped out in all tissues used.

## 4. Discussion

Mesh implants with anchoring systems require a certain holding capacity to be effective [1, 2]. This study provides to a great extent an objective comparison of the holding capacity of four different AS in an experimental setting. Testing of pull-out forces in the porcine pelvic floor as well as in the rectus fascia reveals significant differences between anchors of different designs and materials. With respect to the instability of the “TFS” system under greater loads, design seems to be more important than material. We included tests of the AS in the minipig's rectus fascia because it is more readily comparable to tight structures in the human pelvis, for example, the arcus tendineus or the sacrouterine ligaments, than to the soft tissue of the porcine pelvic floor. Although there are notable differences in tissue strength between the mesentery and the broad ligament in the pelvis, on the one hand, and the rectus fascia, on the other hand, testing in different tissues of the animal model leads to very similar findings. Results only differ for the “TFS” system, with pull-out forces in the broad ligament being comparable to those of “Surelift” and “PelFix,” but revealing reduced pull-out forces when tested in the abdominal wall. With forces above 15 N, the “TFS” AS loses its structural stability.

Our results in the animal model are confirmed by the tests of the different AS in a technical model, a set-up especially designed for these experiments [10]. By means of photo-elasticity, a low pull-out force can be related to a high shearing force in the proximity of the anchor. As expected, these forces concentrate near prominent edges of the anchors and these points of maximum local stress are most likely the predilection sites for rupture. In vivo, this leads to local tissue disruption and thus promotes migration and dislocation of the anchor.

In this setting, the best anchors with excellent pull-out forces are “Surelift” (Neomedic) and “PelFix” (FEG). The “MiniArc” anchor (AMS) and the “TFS” anchor (TFS Surgical) reveal low pull-out forces and are easily prone to dislocation. With regard to the shape of the anchors, we found that, with the higher strain required in the fascia, the arms of the “TFS” easily developed deflections but showed acceptable results with the lower strain used on the porcine pelvic floor as well as in ballistic gelatine. Although “PelFix” shows good results in the animal model as well as in ballistic gelatine, the placement of the “PelFix” AS turned out to be difficult since this mainly two-dimensional anchor has to be placed perpendicular to the muscle fibres. “PelFix” anchors do not remain in place when the anchor is placed parallel to the muscle fibers. This might cause a problem in the clinical setting rather than in an experimental setting. Exact positioning of the anchor with regard to the direction of the muscle fibres is nearly impossible during surgery and can rarely be done in a controlled way. In this regard, “PelFix” may be less suitable for practice. The three-dimensionally designed “Surelift” anchor is more likely to remain in a stable position once it is inserted. Moreover, “Surelift” is small in size (length of less than 1 cm) and thus causes comparably little tissue damage.

We do not exactly know what degree of tension the structures bear in daily life, during the surgical procedure itself, and during the early postoperative phase, for example, coughing when waking from anesthesia. This can hardly be measured. Furthermore, a variability in the mode of anchor placement between different surgeons has to be considered.

Diffusion tensor imaging (DTI) in three-dimensional pelvic floor magnetic resonance imaging as recently introduced by Zijta et al. might offer a better insight into the relationship of forces of the pelvic floor muscles and ligaments in the future [11]. We know that the fascia of the abdominal wall can withstand forces of >20 N. Measurements of the maximum physiological stress caused by abdominal pressure indicate an abdominal wall tension of 16 N/cm for small defects or 32 N/cm for large defects [12]. Future research should give us a better understanding of the mechanical requirements for pelvic floor surgery.

Photoelastic analysis helps to evaluate local tensions in a tissue model and offers a possibility to associate these with local tensions in human tissue that can hardly be measured otherwise. Dense lines indicate high local shearing forces which may lead to dislocation. Thus, we can differentiate between various forms of AS and optimize geometric forms of anchors in order to eliminate excessive local tensions.

Since we cannot test and compare different AS in humans, we have to consider alternatives. Cadaveric testing is not an ideal alternative since the rapid postmortem changes of physicochemical properties of the tissue have to be considered. Regarding various animal models, the tissue features of the pig are most easily comparable to those of the humans in many aspects. Unfortunately, this is largely not true for the area of the pelvic floor. The connective tissue is not as strong as in humans, and some of the basic structures like the arcus tendineus and the sacrouterine ligaments seem to be missing in the tetrapod vertebrates. Therefore, we chose the rectus fascia to serve as a holding structure. The testing of pull-out forces in tissues of different strengths reveals that differences between the various AS remain quite similar provided that the AS keep their structural stability. Additional tests of the different AS in ballistic gelatine confirmed the reproducibility of our data and provided further insight into the deformation of anchors under certain forces. Thus, the potential weaknesses of the available anchoring systems can be assessed.

## 5. Conclusions

Failure of adequate anchor fixation may lead to early dislocation of the devices used for incontinence as well as for prolapse surgery. This might especially be true for minislings or smaller meshes for prolapse repair. These newly developed devices rely on a stable intracorporeal fixation particularly during the procedure and in the early postoperative phase. A comparison of four different AS in an experimental setting reveals significant differences in their holding capacity. Relative differences in holding forces of the AS remained stable independent of being tested in different tissues or ballistic gelatine. The methods of testing introduced here will help to adapt AS to the special requirements needed. The development of experimental methods to test different mechanical aspects is essential before using new devices in patients. Although there may be a high level of confirmation during experimental testing, a thorough follow-up of operated patients is mandatory to make sure that clinical results are consistent with experimental data.

## Figures and Tables

**Table 1:** Pull-out forces of anchorage in ballistic gelatine, in the porcine pelvic floor tissue, and in the rectus fascia.

Anchoring system	Gelatine			Pelvic floor tissue			Rectus fascia		
	*F* [*N*] (mean)	*F* [*N*] (range)	*F*/*F*_PelFix_ [/]	*F* [*N*] (mean)	*F* [*N*] (range)	*F*/*F*_PelFix_ [/]	*F* [*N*] (mean)	*F* [*N*] (range)	*F*/*F*_PelFix_ [/]
MiniArc	4.17	3.86–4.87	0.53	4.73	3.9–5.8	0.37	20.65	5.7–≥50.0	0.41
Surelift	7.10	6.14–8.24	0.91	10.86	6.6–15.2	0.85	39.67	21–≥50.0	0.79
TFS	8.16	7.34–9.08	1.04	11.80	8.3–14.8	0.93	18.74	10.8–27.4	0.37
PelFix	7.81	6.91–8.95	1.00	12.73	8.4–15.6	1.00	50.00	>50.0	1.00

**Table 2:** Significance of the measurements in the different AS.

Anchor			*P *
MiniArc	Rectus	Pelvic floor	0.140
		Gelatine	0.050
	Pelvic floor	Rectus	0.140
		Gelatine	1.000
	Gelatine	Rectus	0.050
		Pelvic floor	1.000

Surelift	Rectus	Pelvic floor	0.001
		Gelatine	0.000
	Pelvic floor	Rectus	0.001
		Gelatine	1.000
	Gelatine	Rectus	0.000
		Pelvic floor	1.000

TFS	Rectus	Pelvic floor	0.071
		Gelatine	0.002
	Pelvic floor	Rectus	0.071
		Gelatine	0.816
	Gelatine	Rectus	0.002
		Pelvic floor	0.816

PelFix	Rectus	Pelvic floor	0.000
		Gelatine	0.000
	Pelvic floor	Rectus	0.000
		Gelatine	0.001
	Gelatine	Rectus	0.000
		Pelvic floor	0.001
